# Molecular and Pathological Analyses of IARS1-Deficient Mice: An IARS Disorder Model

**DOI:** 10.3390/ijms24086955

**Published:** 2023-04-09

**Authors:** Masaki Watanabe, Koya Shishido, Nao Kanehira, Koki Hiura, Kenta Nakano, Tadashi Okamura, Ryo Ando, Hayato Sasaki, Nobuya Sasaki

**Affiliations:** 1 Laboratory of Laboratory Animal Science and Medicine, School of Veterinary Medicine, Kitasato University, 35-1 Higashi-23, Towada 034-8628, Japan; 2 Department of Laboratory Animal Medicine, Research Institute, National Center for Global Health and Medicine, Tokyo 162-8655, Japan; 3 Laboratory of Veterinary Pathology, School of Veterinary Medicine, Kitasato University, 35-1 Higashi-23, Towada 034-8628, Japan

**Keywords:** mitochondrial diseases, hepatic triglyceride, serum ornithine carbamoyltransferase, weak calf syndrome

## Abstract

Most mitochondrial diseases are hereditary and highly heterogeneous. Cattle born with the V79L mutation in the isoleucyl-tRNA synthetase 1 (IARS1) protein exhibit weak calf syndrome. Recent human genomic studies about pediatric mitochondrial diseases also identified mutations in the IARS1 gene. Although severe prenatal-onset growth retardation and infantile hepatopathy have been reported in such patients, the relationship between IARS mutations and the symptoms is unknown. In this study, we generated hypomorphic IARS1^V79L^ mutant mice to develop an animal model of IARS mutation-related disorders. We found that compared to wild-type mice, IARS^V79L^ mutant mice showed a significant increase in hepatic triglyceride and serum ornithine carbamoyltransferase levels, indicating that IARS1^V79L^ mice suffer from mitochondrial hepatopathy. In addition, siRNA knockdown of the IARS1 gene decreased mitochondrial membrane potential and increased reactive oxygen species in the hepatocarcinoma-derived cell line HepG2. Furthermore, proteomic analysis revealed decreased levels of the mitochondrial function-associated protein NME4 (mitochondrial nucleoside diphosphate kinase). Concisely, our mutant mice model can be used to study IARS mutation-related disorders.

## 1. Introduction

Mitochondrial diseases are genetically and phenotypically diverse energy deficiency disorders related to reduced mitochondrial oxidative phosphorylation [1]. Mitochondrial diseases are clinically heterogeneous, can occur at any age, and have a wide range of clinical symptoms. These diseases are rare with an incidence of about 1 in 5000 births [2]. The most common symptoms of mitochondrial diseases are tissue-associated, requiring high production of cellular ATP for proper functioning, affecting the nervous system, heart, skeletal muscle, and liver [3,4]. Therefore, multisystem disorders are diagnostic criteria for mitochondrial diseases [5]. For instance, mitochondrial hepatopathy involves liver damage or failure due to abnormalities in the mitochondrial respiratory chain complex [6].

Isoleucyl-tRNA synthetase 1 (IARS1) is a member of the aminoacyl-tRNA synthetase (AARS) family of enzymes. These enzymes catalyze esterification reactions to link amino acids to cognate tRNAs. For instance, IARS1 catalyzes the ligation of isoleucine to its cognate tRNA consuming ATP [7].

Hirano et al. (2012) identified a homozygous missense mutation (c.235G > C, p.V79L) in exon 3 of the IARS1 gene as a molecular cause of weak calf syndrome in Japanese black cattle (Wagyu). This mutant IARS1 enzyme has 40% less activity than that of the wild-type enzyme [8]. The affected calves exhibit prenatal-onset growth retardation, severe muscle weakness with astasia, and fatty degeneration of liver cells [8,9]. Notably, recent genomic analyses of mitochondrial diseases in children also identified similar mutations in the IARS1 gene [10]. In humans with IARS disorder, the occurrence of bi-allelic mutations such as p.Arg254* and p.Pro437Leu, along with p.Arg739Cys and p.Phe556Ser, have been identified as causative factors of IARS disorder [10,11]. Such child patients suffered from prenatal-onset growth retardation, intellectual disability, muscular hypotonia, hepatopathy with fibrosis and steatosis, diabetes mellitus, and sensorineural hearing loss [10]. IARS1-related disorders are thought to impair protein synthesis due to slowed translation, resulting in mitochondrial dysfunction. However, the detailed mechanism is largely unknown.

Here, we developed a mice model for analyzing IARS1-mutation-related disorders. The hypomorphic IARS1^V79L^ mutant mice had fatty liver at the preweaning age (2 weeks old). In addition, we investigated the mechanism of IARS hypofunction causing liver damage. Our mutant mice model can aid the basic research on IARS-related disorders.

## 2. Results

### 2.1. Introduction of the V79L Mutation Using CRISPR/Cas9

**Figure 1:**
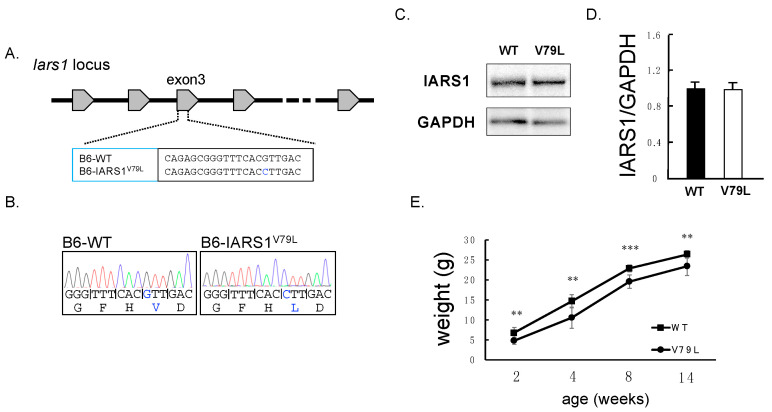
Generation of IARS1^V79L^ mice using a CRISPR/Cas9 system. (**A**) Single-nucleotide polymorphism (G235C) and amino acid substitution (V79L) are illustrated. (**B**) DNA sequencing electropherogram at codon 79 of IARS in B6 and IARS1^V79L^ mice. (**C**,**D**) Western blot analysis showing no deference in IARS1 in B6 and IARS1^V79L^ mice (*n* = 3). (**E**) The temporal fluctuations in the body weight of B6 (*n* = 7) and IARS1^V79L^ (*n* = 7) male mice were monitored and recorded. ** denotes *p* < 0.01. *** denotes *p* < 0.001.

Different bi-allelic mutations in the IARS region have been detected in human IARS disorder, and the specific mutations’ effects on enzyme activity have not been explored. In cattle, Hirano et al. have established that the homozygous V79L mutation leads to the disease and reduces enzyme activity by 40%, prompting us to classify the p.V79L mutation as a hypomorphic mutation in IARS [8]. Thus, the V79L mutation was introduced into the IARS1 gene of B6 mice using the CRISPR/Cas9 system. The desired mutation in the IARS1 gene was confirmed by DNA sequencing having the single nucleotide substitution (c.235G > C) (Figure 1A,B). Additionally, the IARS1^V79L^ protein level in mutant mice was examined by Western blotting, showing no significant difference from the wild-type mice (Figure 1C,D). The only discernible phenotypic difference between the IARS1^V79L^ and wild-type mice was a significant reduction in the body weights of mutant mice at 2, 4, 8, and 14 weeks (Figure 1E). These results indicate that IARS1 hypofunction induced growth retardation in mutant mice.

### 2.2. Histopathological Observation of Mice Liver

**Figure 2:**
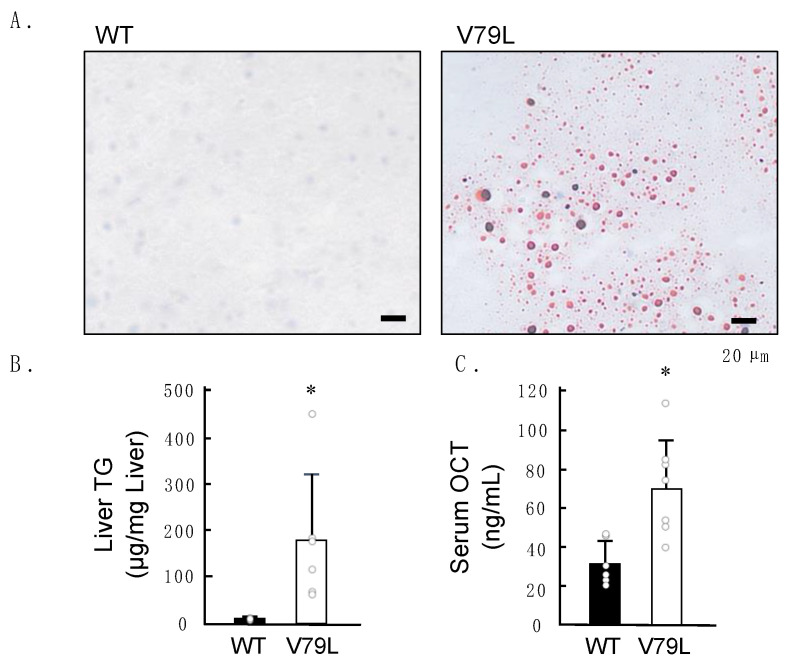
Fatty liver with mitochondrial injury in IARS1^V79L^ mice. (**A**) Oil-red-O-stained liver sections from B6 and IARS1^V79L^ mice and (**B**) quantification of the liver triglyceride and (**C**) serum ornithine carbamoyltransferase levels in B6 (*n* = 6) and IARS1^V79L^ (*n* = 6) mice; * denotes *p* < 0.05.

A whole-body pathological examination of the 16-week-old mice was conducted for phenotypic screening using hematoxylin–eosin (H&E) staining; however, no morphological abnormalities were observed in all specimens (*n* = 5). Since IARS disorder is characterized by the presence of fatty liver from an early age [9], we collected the livers of the 2-week-old male mice and evaluated them for the presence of fat droplets using oil red O (ORO) staining. Three out of thirteen IARS1^V79L^ mice showed ORO-positive fat droplets in their livers (Figure 2A). However, we did not find any liver failure in 16-week-old mutant mice, possibly due to the limited penetrance of the mutant gene.

### 2.3. Elevation of Hepatic Triglycerides and Serum Ornithine Carbamoyltransferase Levels in Mutant Mice

We extracted triglycerides (TGs) from the liver tissues of the 2-week-old male mice. TG contents in the liver of IARS1^V79L^ mice exhibited large variation and were higher than those in the wild-type mice (Figure 2B). Only one out of six IARS1^V79L^ mice showed, by far, the highest content of hepatic TG (450 µg/mg of liver tissue). This can be attributed to the low penetrance of fatty liver in IARS1^V79L^ mice. Ornithine carbamoyltransferase (OCT), a mitochondrial matrix urea cycle enzyme, is primarily expressed in hepatocytes. Animal models of toxicant-induced acute hepatic injury have elevated serum levels of OCTs [12,13]. Consistently, in this study, too, serum OCT levels were significantly higher in 2-week-old IARS1^V79L^ mice than in wild-type mice (Figure 2C). These results indicate that mitochondrial damage caused fatty liver/hepatocytes in IARS1^V79L^ mice.

### 2.4. Downregulation of IARS1 Induces Mitochondrial Dysfunction in HepG2 Cells

**Figure 3:**
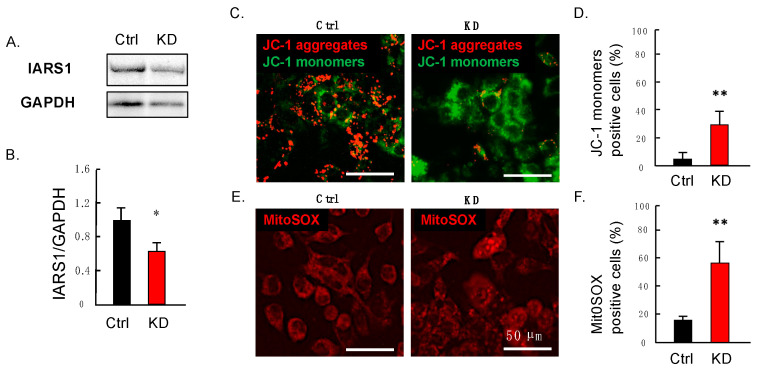
Analysis of IARS1 mitochondrial function in vitro. (**A**,**B**) Western blotting showed significant reductions in IARS1 protein levels in HepG2 cells after transfection with IARS siRNA (*n* = 3). (**C**,**D**) IARS1 knockdown (IARS1-KD) cells showed a decrease in mitochondrial membrane potential (*n* = 3) and (**E**,**F**) an increase in mitochondrial superoxide radicals; * denotes *p* < 0.05. ** denotes *p* < 0.01.

We examined the effect of IARS1 on mitochondrial function in hepatocytes by performing siRNA knockdown (KD) of the IARS1 gene in the hepatocarcinoma-derived cell line HepG2. HepG2 cells are widely used as an in vitro model to investigate the mechanisms underlying fatty liver diseases [14,15]. The IARS1 siRNA-transfected cells had significantly decreased expression of IARS1 compared to the negative control siRNA-transfected cells (Figure 3A,B). The HepG2 cells were incubated with the cationic dye JC-1, which specifically localizes to mitochondria and emits red or green fluorescence, indicating the intact or lost mitochondrial membrane potential, respectively. The control cells showed a red-dotted staining pattern, while the IARS1-KD cells showed a decrease in red fluorescence with a significant increase in green fluorescence cells (Figure 3C,D). Additionally, we used another fluorescent probe named mtSOX Deep Red to detect mitochondrial superoxide radicals. Notably, mitochondria are the major production site of reactive oxygen species (ROS), and their production increases in mitochondrial disorders [16,17,18]. The number of cells with mtSOX Deep Red staining was significantly increased in the IARS1-KD cells (Figure 3E,F), indicating higher levels of ROS.

### 2.5. Administration of Palmitic Acid Increased Fat Droplet Formation in HepG2 Cells

**Figure 4:**
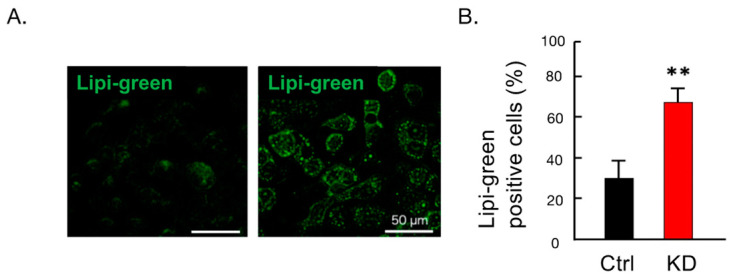
Increased fat accumulation in IARS1-KD HepG2. (**A**,**B**) Palmitic-acid-stimulated intracellular accumulation of fat droplets was significantly higher in IARS1-KD cells (*n* = 3); ** denotes *p* < 0.01.

To assess whether IARS1 hypofunction affects fat accumulation in HepG2 cells, the cells were treated with palmitic acids for 24 h. Compared with control cells, the IARS1-KD cells formed larger and brighter fat droplets (Figure 4A,B), indicating their susceptibility to fat accumulation. This indicates that IARS1 hypofunction causes fatty liver.

### 2.6. Proteomic Analysis

**Figure 5:**
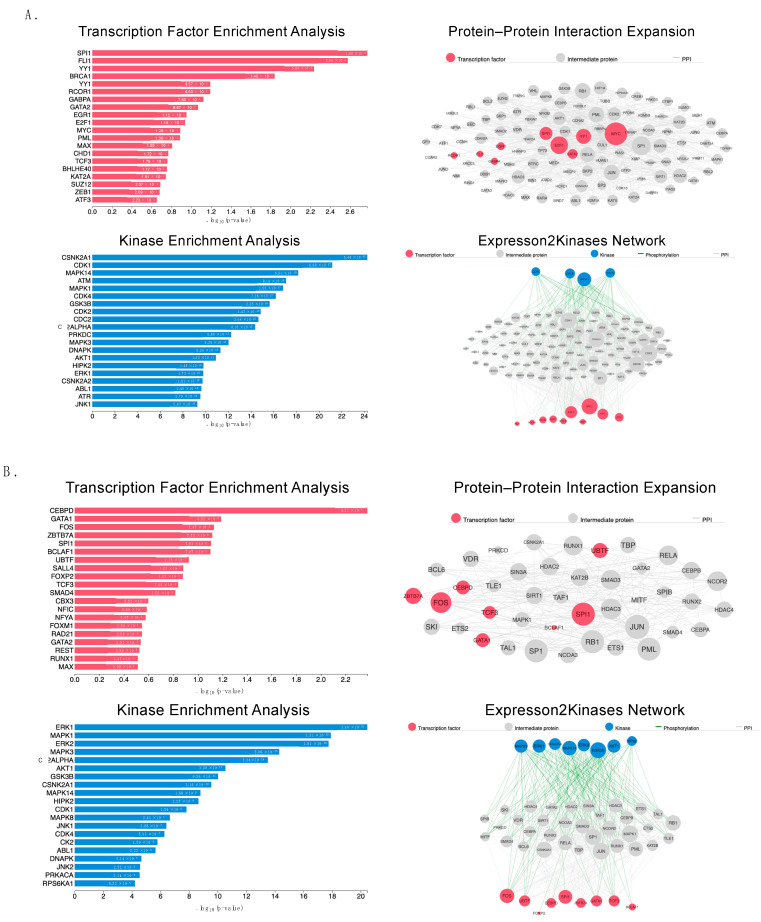
Proteomic analysis of liver samples. Upstream regulatory network of (**A**) upregulated and (**B**) downregulated protein signatures in liver samples from 2-week-old B6 and IARS1V79L mice. Three liver samples each from both mice (*n* = 3) were pooled for analysis. The X2K protein network analysis depicts transcription factors (TFs, red nodes), intermediate proteins (gray nodes), and kinases (blue nodes). Gray edges indicate protein–protein interactions (PPI), and green edges indicate kinase-driven phosphorylation events. Node sizes are relative to expression levels.

To understand the molecular mechanisms of mitochondrial hepatopathy caused by IARS1 hypofunction, we performed proteomic analysis on the isolated livers of 2-week-old male mice. In total, 7048 quantifiable proteins were identified, including 108 differentially regulated proteins between the IARS1V79L and control mice: 42 proteins were upregulated and 66 proteins were downregulated (Table 1 and Table S1). The other remaining proteins showed little change (0.75–1.25-fold change). The Expression2Kinases (X2K) web portal was used to identify the transcription factors (TFs), protein–protein interactions, and kinases responsible for the observed changes in protein expression levels. X2K combines known protein–protein interactions, constructs a network between identified TFs, and predicts upstream regulators [19]. We found that the upregulated differential proteins were significantly associated with TFs, such as SPI1, GATA2, and MYC, and kinases, such as CDK1, ATM, and PRKDC (Figure 5A). On the contrary, the downregulated differential proteins were significantly associated with TFs, such as CEBPD, GATA1, and FOXP2, and kinases, such as ERK1, MAPK1, and AKT1 (Figure 5B).

## 3. Discussion

IARS disorder is an orphan disease in humans, and only a few cases have been reported so far [10]. Weak calf syndrome in livestock has been linked to the IARS1^V79L^ mutation. However, since cattle with IARS-related disorders are culled, it extremely limits the knowledge about this disorder. Therefore, here, we generated IARS1^V79L^ mice that can be exploited as an animal model to study IARS-related disorders in cattle and humans.

IARS1^V79L^ mice had lower body weight and developed fatty liver at low penetrance from an early age. Many findings have linked the mutations in AARS enzymes to human disorders. For instance, mutations in glycyl-tRNA synthetase (GARS) cause autosomal dominant Charcot–Marie–Tooth disease type 2D and neuropathy, and distal hereditary motor, Va type [26]. Autosomal recessive mutations in methionyl-tRNA synthetase (MARS) cause interstitial lung and liver disease; mutations in leucyl-tRNA synthetase (LARS) cause infantile acute liver failure syndrome type 1 [27,28]. Notably, both MARS- and LARS-associated diseases are characterized by liver failure in infancy or early childhood. Although exact liver pathologic features are not clear, significant growth retardation is commonly observed in IARS disorders affecting humans and cattle [9,10]. Considering the lack of cases of IARS disorders and the low penetration of fatty liver in IARS1^V79L^ mice, we still believe that the IARS1^V79L^ mice model partially mimics the IARS disorder in humans and cattle.

Fatty liver since childhood is also common in patients with mitochondrial hepatopathy [6]. Interestingly, IARS1^V79L^ mice showed a significant increase in serum OCT levels, indicating that IARS hypofunction induces mitochondrial hepatopathy [12]. Moreover, IARS1-KD HepG2 cells showed a decrease in mitochondrial membrane potential and increased ROS production, indicating mitochondrial dysfunction. In addition, these cells had a significant accumulation of lipid droplets upon palmitic acid administration. Notably, mitochondrial dysfunction increases oxidative stress and induces abnormal lipid metabolism [29]. Therefore, these results suggest that IARS hypofunction impairs mitochondrial function, causing fatty liver.

Finally, we performed a proteomic analysis of liver samples to elucidate the mechanism behind AARS-associated disease. Our proteomic analysis was based on the hypothesis that decreased isoleucyl-tRNA levels would lead to a global decrease in protein synthesis, causing mitochondrial dysfunction. Surprisingly, 108 of the total 7048 identified proteins were changed sharply, and their alterations were apparently “all or none”; 42 proteins were upregulated and 66 proteins were downregulated between the IARS1^V79L^ and wild-type mice. AARS enzymes have acquired various functions in addition to their canonical tRNA aminoacylation activity [30]. These non-canonical functions are the regulation of gene expression, RNA splicing, tumorigenesis, angiogenesis, and immune response [31,32]. For instance, allergen-induced phosphorylation of lysyl-tRNA synthetase (KARS) triggers its release from the multi-AARS complex, induces conformational changes, and suppresses its translational activity while enhancing the orthogonal function of activating the transcription of immune genes [33,34]. Therefore, the 108 differential proteins in IARS1^V79L^ mice may arise from the unknown failed non-canonical functions of IARS due to the V79L mutation.

Among the sharply downregulated proteins in IARS1^V79L^ mice, we identified a crucial mitochondrial function-related protein, named mitochondrial nucleoside diphosphate kinase (NME4), a member of the multifunctional NDPK/NME protein family. NME4 is predominantly localized to the mitochondrial intermembrane space and binds to the inner membrane by anionic phospholipids such as cardiolipin [35,36]. Mutation/depletion of NME4 impairs mitochondrial structure and function [23,24]. Thus, the downregulation of NME4 may be a key determinant of mitochondrial hepatopathy in IARS1^V79L^ mice. Other sharply downregulated proteins in IARS1^V79L^ mice were fatty-liver-related proteins: AK6, JAK3, LILRB3, and UBASH3B [20,21,22]. Among them, JAK3 may be the one causing fatty liver in IARS1^V79L^ mice, as its knockout is known to cause fatty liver in mice [21,37]. The link between the identified differential proteins and IARS1 should be further investigated to clarify the pathophysiology of IARS-related disease. IARS1 mutations were shown to upregulate the substrates of kinases, such as CDK1, ATM, and PRKDC, which are associated with DNA repair mechanisms [38,39,40,41,42,43]; this could be related to ROS-induced DNA damage.

**Figure 6:**
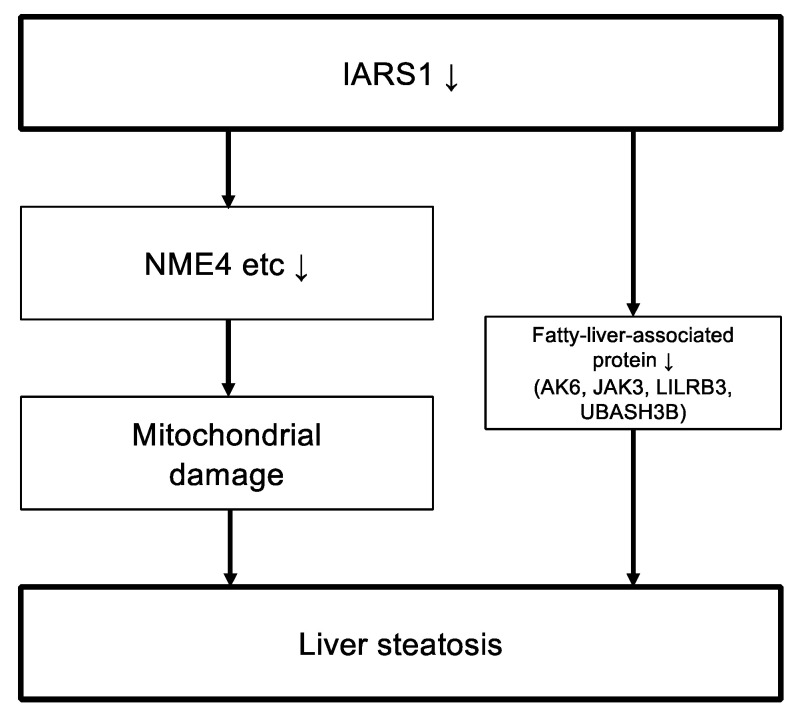
The proposed mechanism of mitochondrial damage and pathogenesis of fatty liver in IARS hypofunction. The downregulation of various mitochondria-associated proteins, including NME4, caused by IARS hypofunction leads to mitochondrial injury and subsequent development of fatty liver. Alternatively, IARS hypofunction may directly contribute to the pathogenesis of fatty liver by downregulating proteins involved in hepatic lipid metabolism, such as AK6, JAK3, LILRB3, and UBASH3B. The arrows (↓) indicate downregulation of protein expression.

In this study, we developed an animal model for the study of IARS disorder. The mice carrying a hypomorphic mutation in IARS1 showed fatty liver at a preweaning age (Table 2). We investigated the mechanisms by which IARS hypofunction causes liver damage (Figure 6). The findings of this study provide new perspectives for basic research on IARS disorder.

## 4. Materials and Methods

### 4.1. Ethical Statements

All animal experiments were performed following the ARRIVE guidelines and the Animal Welfare and Management Act of Japan. Additionally, all experiments were conducted in compliance with the Regulations for the Care and Use of Laboratory Animals at Kitasato University, Japan. The animal experimental protocol was approved by the President of Kitasato University based on the judgment of the Institutional Animal Care and Use Committee of Kitasato University (approval ID: 19–152).

### 4.2. Animals

IARS1^V79L^ mice were generated by CRISPR/Cas9-mediated genome editing. Briefly, the crRNA guiding sequence (5′-TGTCAACGTGAAACCCGCTC-3′) for exon 3 of IARS (NCBI accession number: NM_172015.3) was designed. Both crRNA and tracrRNA were synthesized by Fasmac, Tokyo, Japan. A single-stranded oligodeoxynucleotide (ssODN) for the targeted insertion (5′-AGATATCGTTACGAGATACGCTCACCAGAGCGGGTTTCACCTTGACAGAAGATTCGGGTG-3′) was synthesized by Eurofins Genomics, Brussels, Belgium; the underlined bases indicate a point mutation causing single amino acid alteration, V79L. The recombinant Cas9 protein (60 ng/μL, New England Biolabs, MA, USA), crRNA (0.61 pmol/μL), tracrRNA (0.61 pmol/μL), and ssODN (100 ng/μL) were delivered into fertilized eggs from C57BL/6JJcl (CLEA Japan, Tokyo, Japan) female mice by microinjection. After overnight culture, two-cell embryos were transferred into pseudopregnant female mice. Offsprings carrying the knock-in mutation were confirmed by DNA sequencing using the following primers: forward, 5′-CGTCCTGGATGTCTGCCATT-3′, and reverse, 5′-GGTTATGCTAACAGCCACACC-3′. The resulting founder mice were backcrossed to B6 mice for three generations. The knock-in heterozygote mice were bred with heterozygotes to produce homozygous mice. The air-conditioned animal facility was maintained at 22 ± 2 °C and 40–60% relative humidity with a 12 h light–dark cycle. A standard laboratory diet, CE-2 (CLEA Japan), and tap water were available to mice ad libitum. The body weights of male mice were measured at 2, 4, 8, and 14 weeks.

### 4.3. Western Blotting

Liver tissues from 2-week-old male WT and IARS1^V79L^ mice were collected and lysed in RIPA buffer. Whole-cell lysates were separated by sodium dodecyl sulfate–polyacrylamide gel electrophoresis and then transferred to a polyvinylidene difluoride membrane (GE Healthcare, Chicago, IL, USA) using a wet transfer system (Bio-Rad, Hercules, CA, USA). The membranes were incubated in Blocking One reagent (Nacalai Tesque, Kyoto, Japan) for 1 h at room temperature to block non-specific binding. The membranes were probed with anti-IARS (GTX131733; Gene Tex, Irvine, CA, USA) antibodies and then incubated with horseradish-peroxidase-conjugated antibodies against rabbit immunoglobulin (#7074; Cell Signaling Technology, Beverly, MA, USA). This was followed by the detection with enhanced chemiluminescence ECL prime Western blotting detection reagents (Cytiva, Marlborough, MA, USA). The blots were imaged using an Omega Lum C imaging system (Gel Co., San Francisco, CA, USA). The band intensity of Western blot was measured by using ImageJ 1.53k software “http://imagej.net/ (accessed on 6 August 2021)”, and changes in the IARS1 level were normalized to the housekeeping protein GAPDH.

### 4.4. Histology

We collected tissue samples of major organs (liver, kidney, lung, and heart), lymphoid system (thymus, spleen, and lymph nodes), and reproductive system (testes, prostate, seminal vesicles, ovaries, uterus, and vagina) from 16-week-old male and female mice. All specimens were fixed in a 10% neutral-buffered formalin solution and embedded in paraffin using conventional techniques. The paraffin sections of 5 µm thickness were stained with H&E. The liver tissues from 2-week-old mice were frozen in Tissue-Tek O.C.T. compound (Sakura Finetek Japan, Tokyo, Japan), and 5 μm thick sections were prepared to examine hepatic lipid accumulation. Liver sections were rehydrated with 60% isopropanol and stained for 15 min in freshly made 0.5% ORO stain (Cat# 09755; Sigma-Aldrich, St. Louis, MO, USA) in isopropanol:ddH_2_O (3:2) solution. The slides were rinsed with 60% isopropanol, counter-stained to identify hepatocyte nuclei with modified hematoxylin for 90 s, submerged in tap water for 3 min, followed by rinsing for 30 s in distilled water, and then were finally mounted with Mount-Quick “Aqueous” (Daido Sangyo, Saitama, Japan).

### 4.5. Estimation of Liver TG Level

Total liver lipids were extracted according to Folch et al. [46]. Briefly, flash-frozen tissue samples (75–100 mg) were weighed, homogenized in 1 mL of methanol, and vortexed, followed by the addition of 2 mL of chloroform, 1 mL of water, and centrifugation at 3000 rpm for 15 min at 4 °C in pre-weighed glass vials. The lower organic phase was transferred to a clean vial, dried at 80 °C, and dissolved in isopropanol to make lipid extracts. The lipid extracts were evaluated using a Hitachi 7180 automatic analyzer (Hitachi, Yokohama, Japan).

### 4.6. Measurement of Serum OCT Levels

The 2-week-old male mice were subjected to intraperitoneal anesthesia using a combination of three anesthetic agents: 0.75 mg/kg medetomidine, 4 mg/kg midazolam, and 5.0 mg/kg butorphanol [47]. Subsequently, 100–200 μL of blood samples were collected from the inferior vena cava. The blood was centrifuged at 1700× *g* for 15 min to obtain the supernatant, which was again centrifuged at 1700× *g* for another 15 min. OCT levels in serum samples were measured by an enzyme-linked immunosorbent assay (ELISA) (Yamasa, Chiba, Japan) following the manufacturer’s protocol.

### 4.7. Cell Culture and Silencing of IARS1 Gene

HepG2 cells were cultured in Dulbecco’s modified Eagle’s medium (Fujifilm Wako Pure Chemical Corporation, Osaka, Japan) containing 10% fetal bovine serum, 100 U/mL penicillin, 100 μg/mL streptomycin, and 1% L-glutamine. Cells were grown at 37 °C in an atmosphere of 5% CO_2_/95% air in a cell culture dish. A stealth small interfering RNA (siRNA) (ID: 11166) against human IARS1 mRNA was purchased from Thermo Fisher Scientific (Waltham, MA, USA), and the negative control siRNA (Mission siRNA SIC-001) was obtained from Sigma-Aldrich (St. Louis, MO, USA). siRNAs were transfected using Lipofectamine 3000 reagent following the manufacturer’s instructions (Thermo Fisher Scientific). After 48 h of transfection, the cells were used for subsequent assays. The silencing of the endogenous IARS1 gene was confirmed by Western blotting.

### 4.8. Estimation of the Mitochondrial Membrane Potential and ROS

The mitochondrial membrane potential was assessed using the JC-1 (Dojindo Molecular Technologies, Tokyo, Japan) staining method according to the manufacturer’s protocols and previous reports. HepG2 cells were seeded in a 12-well plate and stained with JC-1 via incubation in the working solution for 20 min.

Mitochondrial superoxide radicals were measured using the MitoSOX^TM^ Red mitochondrial superoxide indicator (YEASEN, Shanghai, China) following the manufacturer’s instructions. The cells were incubated with 5 M MitoSOX ^TM^ Red for 10 min at 37 °C and washed with warm PBS 3 times. All images were obtained with a Floid Cell Imaging Station (Thermo Fisher Scientific, USA).

### 4.9. Treatment with Palmitic Acid

Hepatocytes were exposed to 200 μM palmitic acids (P1383, Sigma-Aldrich) conjugated to fatty-acid-free bovine serum albumin (A8806; Sigma-Aldrich) for 24 h. An amount of 200 µM palmitic acid conjugated to fatty-acid-free bovine serum albumin solution was prepared, as described below. One 100 mM palmitic acids stock solution was prepared in DMSO by heating at 70 °C in a shaking water bath. In an adjacent water bath at 55 °C, a 10% (*w/v*) fatty-acid-free bovine serum albumin solution was prepared in H_2_O. An amount of 10 mM palmitic acid containing 10% bovine serum albumin was diluted in the culture medium to obtain the desired final concentrations. The palmitic acid conjugated to fatty-acid-free bovine serum albumin solution was sterile-filtered through a 0.45 μm pore membrane filter and stored at −20 °C. The accumulated lipid droplets were detected using Lipi-Green staining (Dojindo Molecular Technologies) according to the manufacturer’s instructions. The cultured cells were incubated with Lipi-Green working solution (0.1 μM in PBS) at 37 °C for 30 min. Stained cells were observed under a Floid Cell Imaging Station.

### 4.10. Proteomics

Frozen livers from 2-week-old male mice were examined by LC/MS using a Q Exactive mass spectrometer (Thermo Scientific) at the National Institutes of Biomedical Innovation, Health and Nutrition, Japan. The experiments were carried out by pooling the livers from three mice of each strain and measuring them once. In addition, the peak intensity was calculated by correcting the MaxLFQ (a generic label-free quantification technology) estimated protein quantification log2 value with the top 500 precursors.

### 4.11. Statistical Analysis

All statistical analyses were performed using the software EZR version 1.61 [48], and the statistically significant differences between the two groups were determined by Student’s *t*-test. Data with *p*-value < 0.05 were considered significantly different. Error bars indicate standard deviation.

## 5. Conclusions

In conclusion, the hypomorphic mutation of IARS1 in mice, established in this study, replicates the pathophysiology of IARS1 disorder, including growth retardation and hepatic steatosis, observed in preweaning humans and cattle. Furthermore, it was experimentally demonstrated that in HepG2 cells, hypofunction of IARS1 leads to impaired mitochondrial function and exacerbates the accumulation of lipid droplets. The proteomics analysis also revealed the possibility that NME4 may be an important determinant in mitochondrial hepatopathy, and IARS hypofunction might directly contribute to the pathogenesis of fatty liver by downregulating proteins involved in hepatic lipid metabolisms, such as AK6, JAK3, LILRB3, and UBASH3B. The findings obtained from this study require further validation in human and cattle IARS disorder but are expected to significantly advance the fundamental research of IARS disorder.

## Figures and Tables

**Table 1:** List of upregulated and downregulated proteins in IARS1^V79L^ mice from the proteomic analysis. NaN indicates quantification or identification criteria not reached. # and $ indicate fatty-liver- and mitochondria-related proteins, respectively.

Intensity WT	Intensity V79L	Protein ID	Protein Name	Gene	Protein Description	Reference
**Upregulated proteins**						
NaN	18.72208977	P70403	CASP	*Cux1*	Protein CASP	
NaN	18.66755104	Q9JMA7	CP341	*Cyp3a41a*	Cytochrome P450 3A41	
NaN	15.12169933	Q9DBB1	DUS6	*Dusp6*	Dual specificity protein phosphatase 6	
NaN	18.00749779	Q920L5	ELOV6	*Elovl6*	Elongation of very long chain fatty acids protein 6	
NaN	14.84197235	Q9DBY0	FOXP4	*Foxp4*	Forkhead box protein P4	
NaN	18.80181503	A2ARV4	LRP2	*Lrp2*	Low-density lipoprotein receptor-related protein 2	
NaN	20.06907272	P02762	MUP2	*Mup2*	Major urinary protein 2	
NaN	18.44208145	Q5FW60	MUP20	*Mup20*	Major urinary protein 20	
NaN	17.10871887	Q80UQ2	RASF6	*Rassf6*	Ras association domain-containing protein 6	
NaN	19.52822304	Q9QXZ6	SO1A1	*Slco1a1*	Solute carrier organic anion transporter family member 1A1	
**Downregulated proteins**						
14.14918327	NaN	Q8VCP8	KAD6	Ak6	Adenylate kinase isoenzyme 6	# [20]
18.82424736	NaN	Q8CJ27	ASPM	Aspm	Abnormal spindle-like microcephaly-associated protein homolog	
14.77607822	NaN	Q60943	I17RA	Il17ra	Interleukin-17 receptor A	
14.45795155	NaN	P23611	IRF8	Irf8	Interferon regulatory factor 8	
16.59717178	NaN	Q62137	JAK3	Jak3	Tyrosine-protein kinase JAK3	# [21]
13.80301094	NaN	P97484	LIRB3	Lilrb3	Leukocyte immunoglobulin-like receptor subfamily B member 3	# [22]
12.92001438	NaN	Q9WV84	NDKM	Nme4	Nucleoside diphosphate kinase, mitochondrial	$ [23,24]
23.71816444	NaN	Q8BZF8	PGM5	Pgm5	Phosphoglucomutase-like protein 5	
15.65983963	NaN	Q9CZR3	TM40L	Tomm40l	Mitochondrial import receptor subunit TOM40B	
12.56750202	NaN	Q8BGG7	UBS3B	Ubash3b	Ubiquitin-associated and SH3 domain-containing protein B	# [25]

**Table 2:** Comparison of three species in IARS disorder. Three species demonstrating clinical symptoms resulting from IARS mutations and sites of IARS mutations are indicated. ND is an abbreviation denoting the absence of detected clinical findings in the species. An asterisk (*) indicates an introduced stop codon; fs indicates a frame shift.

	Human	Bovine	Mouse
**Clinical features**			
Growth retardation Hepatopathy with steatosis Muscular hypotonia Intellectual disability Diabetes Immunodeficiency	+ + + + + ND	+ + + ND ND +	+ (Male only) + ND ND ND ND
**Mutation**	p.[R254*]/[P437L] heterozygous [10] p.[R418*]/[I1174N] heterozygous [10] p.[V370G]/[N992D] heterozygous [10] p.[R739C]/[F556S] heterozygous [11] p.[Q671fs]/[T69I] heterozygous [44] p.[L234P]/[R519C] heterozygous [45]	p.[V79L] homozygous [8]	p.[V79L] homozygous [The present study]

## Data Availability

The detailed data of the current study are available from the corre-sponding authors upon reasonable request.

## Supplementary Materials

The supporting information can be downloaded at: https://www.mdpi.com/article/10.3390/ijms24086955/s1.

## References

[1] McFarland R., Taylor R.W., Turnbull D.M. (2010). A neurological perspective on mitochondrial disease. Lancet Neurol..

[2] Skladal D., Halliday J., Thorburn D.R. (2003). Minimum birth prevalence of mitochondrial respiratory chain disorders in children. Brain.

[3] Mccormick E.M., Zolkipli-Cunningham Z., Falk M.J. (2018). Mitochondrial disease genetics update. Curr. Opin. Pediatr..

[4] McBride H.M., Neuspiel M., Wasiak S. (2006). Mitochondria: More than just a powerhouse. Curr. Biol..

[5] Hanaford A.R., Cho Y., Nakai H. (2022). AAV-vector based gene therapy for mitochondrial disease: Progress and future perspectives. Orphanet. J. Rare Dis..

[6] Ayers M., Horslen S.P., Gómez A.M., Squires J.E. (2022). Mitochondrial hepatopathy. Clin. Liver Dis..

[7] Yao P., Fox P.L. (2013). Aminoacyl-tRNA synthetases in medicine and disease. EMBO Mol. Med..

[8] Hirano T., Kobayashi N., Matsuhashi T., Watanabe D., Watanabe T., Takasuga A., Sugimoto M., Sugimoto Y. (2013). Mapping and exome sequencing identifies a mutation in the IARS gene as the cause of hereditary perinatal weak calf syndrome. PLoS ONE.

[9] Ogata Y., Nakao T., Takahashi K., Abe H., Misawa T., Urushiyama Y., Sakai J. (1999). Intrauterine growth retardation as a cause of perinatal mortality in Japanese black beef calves. Zentralbl. Veterinarmed. A.

[10] Kopajtich R., Murayama K., Janecke A., Haack T., Breuer M., Knisely A.S., Harting I., Ohashi T., Okazaki Y., Watanabe D. (2016). Biallelic IARS mutations cause growth retardation with prenatal onset, intellectual disability, muscular hypotonia, and infantile hepatopathy. Am. J. Hum. Genet..

[11] Orenstein N., Weiss K., Oprescu S.N., Shapira R., Kidron D., Vanagaite-Basel L., Antonellis A., Muenke M. (2017). Bi-allelic IARS mutations in a child with intra-uterine growth retardation, neonatal cholestasis, and mild developmental delay. Clin. Genet..

[12] Murayama H., Ikemoto M., Fukuda Y., Tsunekawa S., Nagata A. (2007). Advantage of serum type-I arginase and ornithine carbamoyltransferase in the evaluation of acute and chronic liver damage induced by thioacetamide in rats. Clin. Chim. Acta.

[13] Murayama H., Ikemoto M., Fukuda Y., Nagata A. (2008). Superiority of serum type-I arginase and ornithine carbamyltransferase in the detection of toxicant-induced acute hepatic injury in rats. Clin. Chim. Acta.

[14] Dongiovanni P., Crudele A., Panera N., Romito I., Meroni M., De Stefanis C., Palma A., Comparcola D., Fracanzani A.L., Miele L. (2020). Β-Klotho gene variation is associated with liver damage in children with NAFLD. J. Hepatol..

[15] Li S., Dou X., Ning H., Song Q., Wei W., Zhang X., Shen C., Li J., Sun C., Song Z. (2017). Sirtuin 3 Acts as a negative regulator of autophagy dictating hepatocyte susceptibility to lipotoxicity. Hepatology.

[16] Indo H.P., Davidson M., Yen H., Suenaga S., Tomita K., Nishii T., Higuchi M., Koga Y., Ozawa T., Majima H.J. (2007). Evidence of ROS generation by mitochondria in cells with impaired electron transport chain and mitochondrial DNA damage. Mitochondrion.

[17] Takashi Y., Tomita K., Kuwahara Y., Roudkenar M.H., Roushandeh A.M., Igarashi K., Nagasawa T., Nishitani Y., Sato T. (2020). Mitochondrial dysfunction promotes aquaporin expression that controls hydrogen peroxide permeability and ferroptosis. Free Radic. Biol. Med..

[18] Wallace D.C., Fan W., Procaccio V. (2010). Mitochondrial energetics and therapeutics. Annu. Rev. Pathol..

[19] Clarke D.J., Kuleshov M.V., Schilder B.M., Torre D., Duffy M.E., Keenan A.B., Lachmann A., Feldmann A.S., Gundersen G.W., Silverstein M.C. (2018). eXpression2Kinases (X2K) Web: Linking expression signatures to upstream cell signaling networks. Nucleic. Acids Res..

[20] Boison D., Scheurer L., Zumsteg V., Rülicke T., Litynski P., Fowler B., Brandner S., Mohler H. (2002). Neonatal hepatic steatosis by disruption of the adenosine kinase gene. Proc. Natl. Acad. Sci. USA.

[21] Mishra J., Verma R.K., Alpini G., Meng F., Kumar N. (2015). Role of janus kinase 3 in predisposition to obesity-associated metabolic syndrome. J. Biol. Chem..

[22] Lu Y., Jiang Z., Dai H., Miao R., Shu J., Gu H., Liu X., Huang Z., Yang G., Chen A.F. (2018). Hepatic leukocyte immunoglobulin-like receptor B4 (LILRB4) attenuates nonalcoholic fatty liver disease via SHP1-TRAF6 pathway. Hepatology.

[23] Boissan M., Dabernat S., Peuchant E., Schlattner U., Lascu I., Lacombe M. (2009). Mammalian Nm23/NDPK family: From metastasis control to cilia movement. Mol. Cell Biochem..

[24] Lacombe M., Lamarche F., De Wever O., Padilla-Benavides T., Carlson A., Khan I., Huna A., Vacher S., Calmel C., Desbourdes C. (2021). The mitochondrially-localized nucleoside Diphosphate kinase D (NME4) is a novel metastasis suppressor. BMC Biol..

[25] Ma M., Xie W., Li X. (2021). Identification of autophagy-related genes in the progression from non-alcoholic fatty liver to non-alcoholic steatohepatitis. Int. J. Gen. Med..

[26] Antonellis A., Ellsworth R.E., Sambuughin N., Puls I., Abel A., Lee-Lin S., Jordanova A., Kremensky I., Christodoulou K., Middleton L.T. (2003). Glycyl tRNA synthetase mutations in charcot-marie-tooth disease type 2d and distal spinal muscular atrophy type V. Am. J. Hum. Genet..

[27] Hadchouel A., Wieland T., Griese M., Baruffini E., Lorenz-Depiereux B., Enaud L., Graf E., Dubus J.C., Halioui-Louhaichi S., Coulomb A. (2015). Biallelic mutations of the methionyl-trna synthetase (MARS) cause a specific type of pulmonary alveolar proteinosis prevalent on réunion island. Am. J. Hum. Genet..

[28] Casey J.P., McGettigan P., Lynam-Lennon N., McDermott M., Regan R., Conroy J., Bourke B., Sullivan J.O., Crushell E., Lynch S. (2012). Identification of a mutation in LARS as a novel cause of infantile hepatopathy. Mol. Genet. Metab..

[29] Begriche K., Igoudjil A., Pessayre D., Fromenty B. (2006). Mitochondrial dysfunction in NASH: Causes, consequences and possible means to prevent it. Mitochondrion.

[30] Pang Y.L.J., Poruri K., Martinis S.A. (2014). tRNA synthetase: tRNA Aminoacylation and Beyond. Wiley Interdiscip Rev. RNA.

[31] Turvey A.K., Horvath G.A., Cavalcanti A.R.O. (2022). Aminoacyl-tRNA synthetases in human health and disease. Front. Physiol..

[32] Smirnova E.V., Lakunina V.A., Tarassov I., Krasheninnikov I.A., Kamenski P.A. (2012). Noncanonical functions of aminoacyl-tRNA synthetases. Biochemistry.

[33] Ofir-Birin Y., Fang P., Bennett S., Zhang H., Wang J., Rachmin I., Shapiro R., Song J., Dagan A., Pozo J. (2013). Structural switch of Lysyl-tRNA synthetase between translation and transcription. Mol. Cell.

[34] Baymiller M., Nordick B., Forsyth C.M., Martinis S.A. (2022). Tissue-specific alternative splicing separates the catalytic and cell signaling functions of human Leucyl-tRNA synthetase. J. Biol. Chem..

[35] Boissan M., Schlattner U., Lacombe M. (2018). The NDPK/NME Superfamily: State of the Art. Lab. Invest..

[36] Li Y., Xu J., Lu Y., Bian H., Yang L., Wu H., Zhang X., Zhang B., Xiong M., Chang Y. (2021). DRAK2 aggravates nonalcoholic fatty liver disease progression through SRSF6-associated RNA alternative splicing. Cell Metab..

[37] Xu Q., Qi W., Zhang Y., Wang Q., Ding S., Han X., Zhao Y., Song X., Zhao T., Zhou L. (2020). DNA methylation of JAK3/STAT5/PPARγ regulated the changes of lipid levels induced by Di (2-Ethylhexyl) phthalate and high-fat diet in adolescent rats. Environ. Sci. Pollut. Res..

[38] Qin L., Fan M., Candas D., Jiang G., Papadopoulos S., Tian L., Woloschak G., Grdina D., Li J. (2015). CDK1 enhances mitochondrial bioenergetics for radiation-induced DNA repair. Cell Rep..

[39] Liao H., Ying S. (2017). CDK1: Beyond cell cycle regulation. Aging.

[40] Sancar A., Lindsey-Boltz L.A., Ünsal-Kaçmaz K., Linn S. (2004). Molecular mechanisms of mammalian DNA repair and the DNA damage checkpoints. Annu. Rev. Biochem..

[41] Guleria A., Chandna S. (2016). ATM kinase: Much more than a DNA damage responsive protein. DNA Repair.

[42] Papeta N., Zheng Z., Schon E.A., Brosel S., Altintas M.M., Nasr S.H., Reiser J., D’Agati V.D., Gharavi A.G. (2010). Prkdc participates in mitochondrial genome maintenance and prevents adriamycin-induced nephropathy in mice. J. Clin. Invest..

[43] Watanabe M., Takahashi Y., Hiura K., Nakano K., Okamura T., Sasaki H., Sasaki N. (2021). A Single Amino Acid Substitution in PRKDC is a Determinant of Sensitivity to Adriamycin-Induced Renal Injury in Mouse. Biochem. Biophys. Res. Commun..

[44] Smigiel R., Biela M., Biernacka A., Stembalska A., Sasiadek M., Kosinska J., Rydzanicz M., Ploski R. (2017). New evidence for association of recessive IARS gene mutations with hepatopathy, hypotonia, intellectual disability and growth retardation. Clin. Genet..

[45] Zou T., Sun H., Zhu Y., He T., Ling W., Zhu H., Lin Z., Liu Y., Liu S., Wang H. (2022). Compound heterozygous variations in IARS1 cause recurrent liver failure and growth retardation in a Chinese patient: A case report. BMC Pediatr..

[46] Folch J., Lees M., Stanley G.H.S. (1957). A simple method for the isolation and purification of total lipides from animal tissues. J. Biol. Chem..

[47] Kawai S., Takagi Y., Kaneko S., Kurosawa T. (2011). Effect of three types of mixed anesthetic agents alternate to ketamine in mice. Exp. Anim..

[48] Kanda Y. (2013). Investigation of the Freely Available Easy-to-use software “EZR” for medical statistics. Bone Marrow Transplant..

